# Selective Optical Imaging for Detection of Bacterial Biofilms in Tissues

**DOI:** 10.3390/jimaging9080160

**Published:** 2023-08-15

**Authors:** Michael Okebiorun, Cody Oberbeck, Cameron Waite, Samuel Clark, Dalton Miller, Elisa H. Barney Smith, Kenneth A. Cornell, Jim Browning

**Affiliations:** 1 Biomedical Engineering Program, Boise State University, Boise, ID 83725, USA; michaelokebiorun@u.boisestate.edu; 2 Department of Electrical and Computer Engineering, Boise State University, Boise, ID 83725, USA or elisa.barney@ltu.se (E.H.B.S.); 3 Department of Mechanical and Biomedical Engineering, Boise State University, Boise, ID 83725, USA; 4 Department of Mathematics, Boise State University, Boise, ID 83725, USA; 5 Department of Chemistry and Biochemistry, Boise State University, Boise, ID 83725, USAkencornell@boisestate.edu (K.A.C.); 6 Autonomous Systems and Software Program, Luleå Tekniska Universitet, 97187 Luleå, Sweden

**Keywords:** biofilm imaging, wounds, trypan blue, image processing, segmentation

## Abstract

Significance: The development of an imaging technique to accurately identify biofilm regions on tissues and in wounds is crucial for the implementation of precise surface-based treatments, leading to better patient outcomes and reduced chances of infection. Aim: The goal of this study was to develop an imaging technique that relies on selective trypan blue (TB) staining of dead cells, necrotic tissues, and bacterial biofilms, to identify biofilm regions on tissues and wounds. Approach: The study explored combinations of ambient multi-colored LED lights to obtain maximum differentiation between stained biofilm regions and the underlying chicken tissue or glass substrate during image acquisition. The TB imaging results were then visually and statistically compared to fluorescence images using a shape similarity measure. Results: The comparisons between the proposed TB staining method and the fluorescence standard used to detect biofilms on tissues and glass substrates showed up to 97 percent similarity, suggesting that the TB staining method is a promising technique for identifying biofilm regions. Conclusions: The TB staining method demonstrates significant potential as an effective imaging technique for the identification of fluorescing and non-fluorescing biofilms on tissues and in wounds. This approach could lead to improved precision in surface-based treatments and better patient outcomes.

## 1. Introduction

Chronic wounds refer to injuries that cannot by themselves achieve anatomical and functional normalcy in a timely and orderly fashion due to various internal and external factors [1]. A recent study estimates the treatment of about 8.2 million people with wounds costs about $96.8 billion. Chronic wounds are, however, mostly seen in the elderly population in the United States with 3% of the population >65 years old with chronic wounds as compared to 2% of the total population [1,2]. Of the several factors that are responsible for the delay in chronic wound healing, bacterial biofilm has been shown to be a major one [2]. 60% of chronic wounds have been found to be associated with biofilms [2]. Diagnosis, prognosis and surface-based treatment of bacterial biofilm infections on tissues, however, often require a mesoscale imaging technique for precision and accuracy [1,2,3,4]. On a microscopic level, biofilms can be visualized using techniques such as scanning electron microscopy, environmental scanning electron microscopy, and helium ion microscopy [5]. Another biofilm imaging technique used is Raman microscopy, which provides a physical and chemical characterization of the biofilm structure and provides information about organic and inorganic constituents of a biofilm [6]. Perhaps the most popular biofilm imaging method is confocal laser scanning microscopy because of its unique ability to produce 3D sample images. However, biofilm imaging on tissues such as chronic wounds, requires imaging at the mesoscale level (>1 mm), which is difficult to achieve outside of laboratory settings with many microscopy-based imaging techniques.

Mesoscale detection methods that have been used to examine biofilms include ultrasound, X-ray, and magnetic resonance imaging techniques [7,8,9,10,11]. Vaidya et al. employed ultrasound imaging to characterize *Haemophilus influenzae* and *Streptococcus pneumoniae* biofilms in children during nasopharyngeal colonization [8]. X-ray tomography imaging has also been shown to be an effective means to capture the morphology of biofilms in a 3D porous media [12]. Optical coherence tomography (OCT) has also been found to be a suitable tool for mesoscale imaging of biofilms [5]. OCT relies on the detection of reflected and scattered light signals to image biofilms on various substrates such as glass slides and polymer membranes [5,13,14,15]. The major drawback to these mesoscale-level imaging techniques is that they do not produce images that clearly distinguish biofilms from the underlying tissue or tissue-like substrate that are necessary to develop precise surface-based treatments of biofilms that occur in a chronic wound. Recently, Jones et al. reported the use of a Moleculight™ imaging device, a fluorescent imaging device that employs the inherent autofluorescence found in most wound bacteria to produce a fluorescence map of bacterial biofilms on the wound surface [16]. *Staphylococcus aureus*, for example, produces red fluorescence upon excitation at 405 nm. One major drawback with this technique as a biofilm detection tool is that many soft tissues also fluoresce at 405 nm, which makes it difficult to distinguish biofilms from adjacent wound tissues. Also, not all bacteria fluoresce which would render this technology unusable in this case. This fluorescent imaging technique would, however, be reproduced as the gold standard for this study.

A number of dyes have been proposed to selectively stain biofilms. Nakagami et al. proposed a blotting technique that used a nitrocellulose membrane to transfer a map of the biofilms on the wound surface that were selectively detected using alcian blue staining [17]. However, transfer of the sample to the nitrocellulose membrane may result in further undesirable spreading of the biofilms over the wound surface. trypan blue (TB) has also been explored as a biofilm selective dye for imaging [18,19,20]. TB was initially used in the 19th century as a textile dye and compound to treat trypanosomal infections [21]. TB has also been explored over the years as a dye to stain tissues during biopsies, or to stain living cells and organisms [18,19,20,21,22,23,24,25]. In an image-guided surgery study, Chang et al. assessed the various concentrations of TB required to effectively stain a rabbit anterior eye capsule and found that a 0.1% TB (*w*/*v*) solution was sufficient to identify the eye capsule [26]. Other work reported that effective TB staining varied from 0.125–0.6% [27,28]. In dye exclusion assays, the ability of TB to penetrate compromised cell membranes of dead cells has long been used to distinguish between live and dead cells in light microscopy [23]. As there are known differences between mammalian and bacterial biofilms in terms of membrane lipid composition, transmembrane potential, the presence/absence of cell walls etc., these differences may translate to exploitable differential TB staining [29]. This knowledge provides a rationale for developing a TB staining procedure (concentration, staining time) that has the potential to selectively stain bacteria biofilms on wound surfaces. Acquisition of stained sample images is usually done with a monochrome camera and selective filters [30]. Monochrome cameras have an advantage over color cameras in that they can capture all the incoming light at each pixel and thus perform better in low lighting conditions and have intrinsically higher frame rates [30]. However, selective filters are required for color feature distinction in the acquired images.

In the present study, the focus lies on developing a cutting-edge imaging technique, explicitly designed for detecting bacterial biofilms on chronic wounds. This method is fundamentally built upon the utilization of trypan blue, a frequently used dye, to stain biofilm samples grown on glass slides and chicken tissue, thus simulating a wound environment effectively. The stained samples undergo imaging through a high sensitivity monochrome camera under a spectrum of colored illuminations. Through the application of a unique image subtraction method, biofilm regions are distinctly highlighted. A comprehensive analysis follows this step, with the fluorescent-based biofilm imaging serving as the validation benchmark. The integration of these diverse techniques aims to introduce a new, efficient method for the accurate detection and analysis of wound biofilms.

## 2. Experimental Setup and Procedures

This study followed a structured approach to develop and validate an imaging technique for bacterial biofilm detection. A total of 18 samples were prepared, with biofilms grown on two types of substrates—12 glass slides and 6 chicken tissue samples. The use of chicken tissue, although not identical to wound tissue, served as a model to demonstrate the feasibility of the proposed method. Following the staining of these samples with trypan blue (TB), the proposed TB imaging technique to acquire images was carried out, followed by validating these using the established fluorescence imaging technique. These images then underwent preprocessing, involving enhancement of biofilm visibility, differentiation of biofilm regions from the substrate through color subtraction, and segmentation of these regions using masks made from both manual and automated thresholding methods, specifically Otsu’s algorithm. The final stage (Validation) involved a comparative analysis of these preprocessed images to ascertain the degree of similarity between the biofilm regions detected by the TB imaging method and those detected by fluorescence imaging. The objective was to evaluate the reliability and accuracy of the proposed TB imaging technique in comparison to the established fluorescence imaging method. Detailed descriptions of each step in this approach are provided in the following subsections.

### 2.1. Sample Preparation

*Pseudomonas fluorescens* 1-day biofilms grown on glass coupons or on chicken breast tissue were employed in this study. Briefly, a *Pseudomonas fluorescens* culture was prepared from a single isolated colony inoculated into 10 mL Luria Bertani (LB) broth and grown overnight at 25 °C. For biofilms grown on glass coupons, sterile 2 cm × 2 cm coverslips were placed vertically in a 12-well plate. Each well of the plate was inoculated with 2 mL fresh LB broth and 0.1 mL overnight culture. The plates were then incubated at 25 °C for 24 h with shaking (170 rpm). Prior to staining, coverslips were dipped three times in sterile phosphate buffered saline (PBS) to remove planktonic cells, and then rubbed with an alcohol wipe to remove biofilm from the edges and one side of the coupon to simplify subsequent image collection. Negative biofilm control samples consisted of glass coverslips that were incubated under similar conditions in sterile LB broth.

For biofilms grown on tissue, fresh chicken breasts were obtained from a local grocery store, cut into approximately 1.2 cm × 1.2 cm × 0.5 cm pieces, and placed in a sterile 150 mm Petri dish. Tissue samples were then washed with sterile deionized water, wiped with sterile paper towels soaked in isopropanol, and briefly air dried in a biosafety cabinet. Tissue samples were placed in individual wells of 24-well plates and immobilized with 0.5 mL 1% agarose in Dulbecco’s Minimal Eagles Medium (DMEM). The center of the chicken sample was inoculated using a cotton swab containing a sample of the *Ps. Fluorescens* overnight culture diluted 1:10 in sterile LB. The inoculated chicken samples were incubated for 24 h at 25 °C without shaking. Negative control samples consisted of chicken tissue samples.

### 2.2. Trypan Blue Staining

The glass coverslips containing 1-day biofilms (and negative controls) were stained by submerging them in 0.4% TB (*w*/*v*) solution in PBS for 20 min. The samples were then rinsed in PBS to remove unbound dye, and the blue-stained biofilms imaged.

To optimize TB staining of biofilms on chicken relative to the background tissue, a preliminary experiment was conducted to evaluate the impact of different TB concentrations (0.01, 0.04, 0.1, 0.4% *w*/*v*) and incubation times (10, 20, 40 min) based on parameters reported in the literature [19,24,28,31]. In all the cases, the chicken samples in 24-well plates were completely submerged in the TB solution for the indicated time span. The TB solution was then aspirated, and the tissue samples rinsed with PBS to remove unbound TB dye. The biofilm regions were expected to be differently stained compared to the background uninoculated tissues. Based on several visual analyses, it was found that staining with 0.04% TB solution for 10 min was sufficient to differentiate stained biofilms from uninoculated chicken tissue.

### 2.3. Imaging Setup

**Figure 1:**
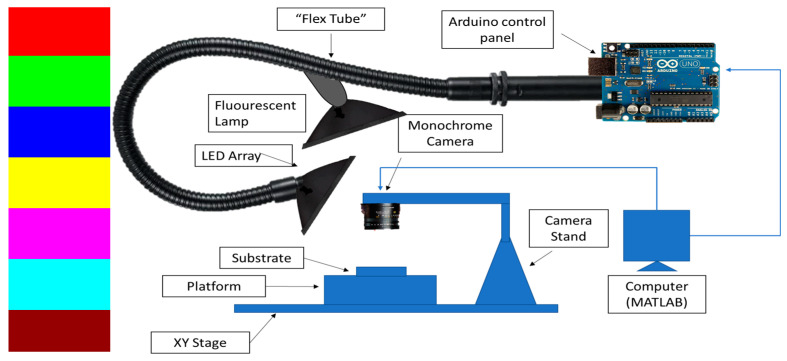
Imaging setup after the sample undergoes trypan blue staining. Image acquisition is carried out using a Mightex monochrome camera viewing samples illuminated under various colored lights for automated segmentation of the biofilm region. (The LED colors explored for image optimization are shown on the left side of the panel).

Imaging was performed using the system described in Figure 1**.** The system is made up of a monochrome camera, a Neopixel 256-LED array capable of producing multi-colored lights, a 405 nm fluorescent lamp, an Arduino Uno, and a MATLAB and Statistics (Toolbox Release 2012b, The MathWorks, Inc., Natick, MA, USA/ LabVIEW 2021, National Instruments Corporation, Austin, TX, USA) that automates the image acquisition and image processing. The Arduino Uno is programmed from MATLAB to make the LED array produce different colored lights for each image capture using the monochrome camera (Figure 1 left side shows the colors explored). The colored lights were explored to obtain a filtering mechanism to selectively pass frequencies specific to the biofilm region relative to the background.

### 2.4. Image Acquisition

#### 2.4.1. Proposed Imaging Technique

Following the staining procedure with trypan blue, a monochrome camera sensor was employed for image acquisition. The decision to use this type of sensor was based on its capacity to deliver greater detail and sensitivity compared to a color sensor. The images obtained are 2D monochromatic images with an intensity range of 0–255, reflecting the 8-bit depth of the 1.3-megapixel camera. To achieve a clear differentiation between the substrate and the stained biofilm regions, a Neopixel LED array coupled with a diffuser was utilized for multicolor illumination. This setup facilitated the production of various colored lights under which the images were illuminated and captured. Each colored light was turned ON sequentially, and an image was acquired at each stage. The specific use of the images captured under different colored lights is further elaborated upon in the thresholding sub-section.

#### 2.4.2. Fluorescence Imaging (Validation Standard)

*Ps. Fluorescens* biofilms are known to contain pyoverdine—a green fluorophore excited with 405 nm light. Therefore, akin to the proposed imaging technique, the image acquisition uses a monochrome camera, a 560–580 nm green filter for emission capture, and a 405 nm UV lamp. Notably, the imaging process in this study is meticulously orchestrated to secure comparable image sets for validation. Specifically, once the samples undergo staining with trypan blue, an automated imaging system, controlled by a custom LabVIEW program, is immediately employed to capture both fluorescent and TB images. This automatic system transitions smoothly between different lights, including fluorescent lamps, ensuring the capture of images under identical conditions. The outcome is a pair of images, FL and TB, both derived from the same sample and under similar conditions, paving the way for direct comparisons in subsequent analysis.

### 2.5. Thresholding

**Figure 2:**
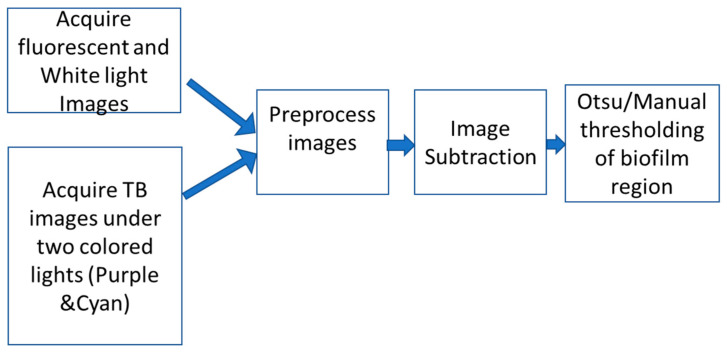
Block diagram of image acquisition and processing for biofilm region detection. Images are acquired, artifacts are removed by glare reduction, noise removal, cropping and contrast enhancement algorithms image subtraction is done, and Otsu/manual thresholding of biofilm region is carried out. Image processing is done in MATLAB.

Both image processing sequences, represented in Figure 2, begin by addressing image artifacts and noise inherent to the camera’s sensor. A Gaussian filter using MATLAB was employed to model the noise, effectively smoothing it out. This noise reduction procedure aids in enhancing the quality and reliability of subsequent image analysis. After noise reduction, the images are cropped to select the region of interest. The cropping is performed manually, focusing on the chicken tissue while excluding extraneous elements, such as the plate or platform on which the sample is placed. This step is vital to ensure that subsequent analysis and computations are focused on the relevant regions of the image.

In the subsequent step, glare or ‘hot pixels’ caused by specular reflections from the illuminating lights are addressed. Such glare appears as pixels with an intensity value of 255 and has the potential to skew image analysis. This is mitigated by substituting the glare-affected pixels with the intensity values of neighboring pixels untouched by reflections. Glare correction aids in further enhancing the fidelity of the image for subsequent processing stages. Following this, a contrast enhancement is performed on the images. The enhancement procedure entails a linear transformation of the image’s pixel intensities, expanding the contrast from its original range to encompass the full intensity range from 0 to 255. This stage is instrumental in improving visibility and distinction between various regions in the image.

In the wake of contrast enhancement, image subtraction is executed for superior segmentation of biofilm regions. The process engages element-wise subtraction of corresponding pixels in images taken under varying illumination conditions. More specifically, images captured under assorted lights are subtracted from those acquired under cyan light (~5.9 × 10^14^ Hz), which provides the most representative view of the biofilm region. Different combinations of color subtraction were probed, and it was found that subtracting the purple-colored image (~7.5 × 10^14^ Hz) from the cyan image yielded the most pronounced differentiation between biofilm regions and the background tissue. For fluorescent imaging, white light images were subtracted from fluorescent images. The image subtraction stage is crucial for distinguishing between stained biofilm regions and the unstained background.

The final stage involves the application of thresholding to produce a mask to further differentiate biofilm regions from the background. Both manual and automatic thresholding techniques were implemented. Manual thresholding involved the selection of the threshold as the lowest intensity value deemed to be from the biofilm region. In the case of automatic thresholding, Otsu’s algorithm (MATLAB ‘Otsuthresh’ function) was utilized. As a variance-based thresholding technique, it iterates through all possible threshold values and measures the spread of pixel levels on each side of the threshold. The optimal threshold value is identified where the sum of the background and foreground (biofilm regions) spreads is minimal. This thresholding technique can be applied to generate a bilevel stain/no stain image or to erase the no-stain region, thus ensuring the image solely highlights the content in the stained region [32].

### 2.6. Validation

**Figure 3:**
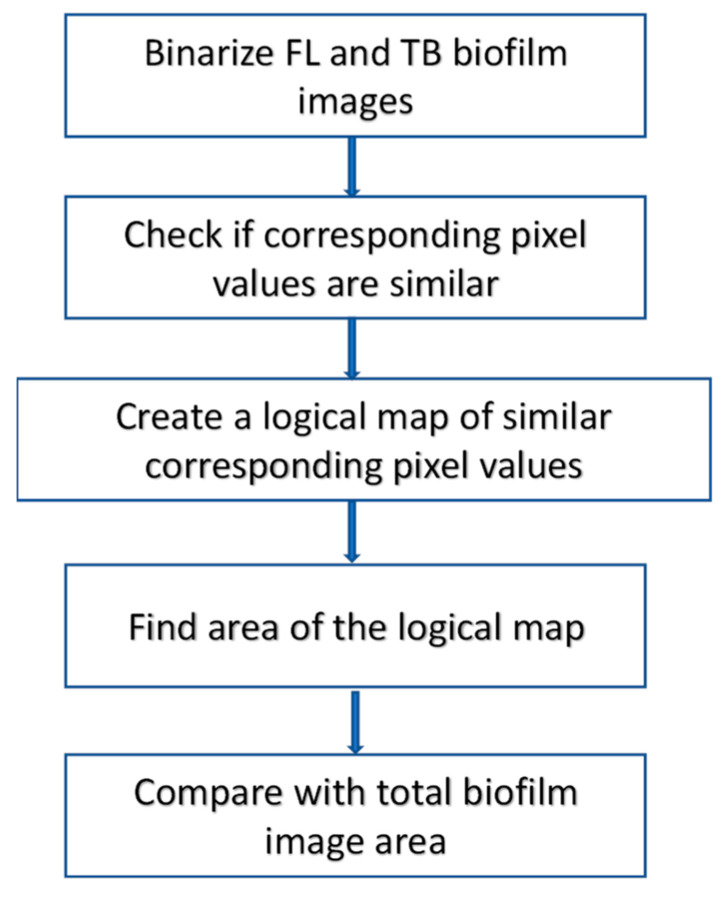
Shape similarity evaluation procedure.

In order to validate images yielded by the proposed technique with the corresponding fluorescent image (validation standard), the morphology of the biofilm regions ascertained by the two methods is compared. Figure 3 delineates the steps involved in achieving the shape similarity values. Once each biofilm difference image undergoes thresholding, resulting in a bilevel image, the negative of the XOR operation is enacted to compare the two images. This operation indicates ‘1′s at pixel locations sharing the same pixel values on both images and ‘0′s otherwise. Consequently, the shape similarity measure is calculated as follows:

(1) $$Shape\;similarity=\frac{Area\;of\;similar\;logical\;pixels}{Total\;area\;of\;logical\;pixels}$$

This gives a metric of similarity since the images were taken under the same physical condition such as location of sample, camera angle etc.

## 3. Experimental Results

This section discusses the results of the trypan blue staining procedure, image acquisition, and image processing results.

### 3.1. Image Processing—Bacterial Biofilms on Glass

**Figure 4:**
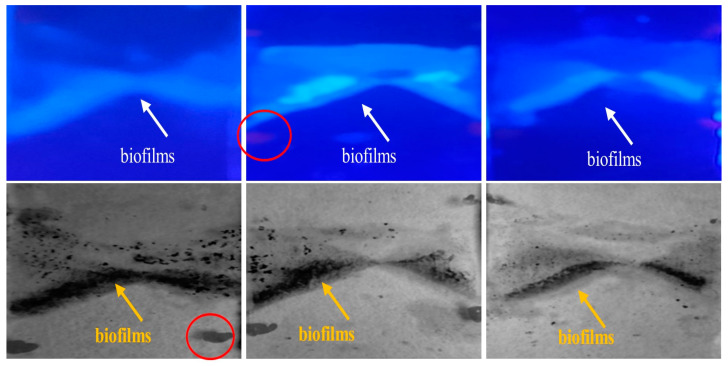
(**top**) Fluorescent images of 3 different biofilm-glass samples with (**bottom**) their corresponding images taken by the proposed method. The images are obtained with a regular color sensor under 405 nm excitation light. The cyan region suggests the presence of biofilms, while the dark region in the bottom images suggest biofilm presence. The red-circled objects are just markers for image alignment purposes.

Figure 4 presents images of glass biofilm samples captured using fluorescence (top) and the proposed trypan blue staining technique (bottom). Both imaging methods reveal similar features, validating the effectiveness of the approach. Importantly, trypan blue does not stain the background glass slides, allowing it to selectively highlight the biofilm distribution on the sample. In these images, the *Ps. fluorescens’* pyoverdine content provides the cyan fluorescence, unveiling the distribution of biofilms on the glass slide. The blue background represents the non-fluorescent areas of the glass slide. In the images obtained with the trypan blue technique, the dark regions indicate the biofilm distribution. The bright background corresponds to the glass slide regions devoid of biofilms. A visual inspection suggests a significant resemblance between the biofilm regions identified by fluorescence and trypan blue techniques.

**Figure 5:**
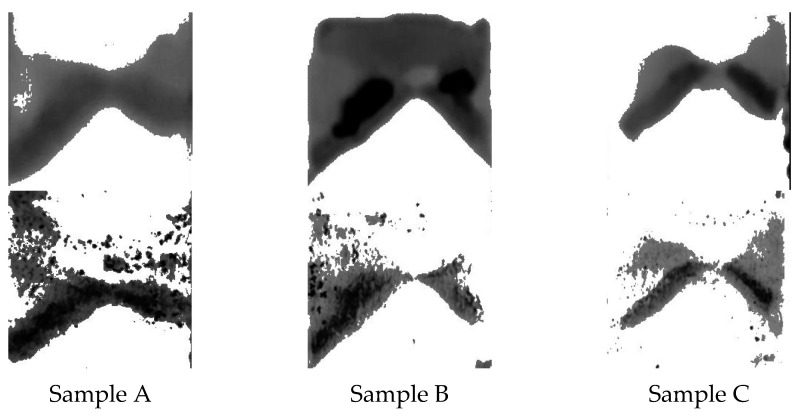
Three sets of biofilm region obtained after Otsu thresholding to remove the non-stained background. Each set contains a fluorescent biofilm image (**top**) and a trypan blue-based image (**bottom**). The darker the region indicate a heavier biofilm presence. The fluorescent images (standard) appear to reveal a more comprehensive biofilm region compared to the TB technique.

Figure 5 showcases the results of the Otsu segmentation technique applied to the biofilm images obtained from both the fluorescence and the trypan blue staining techniques for three samples in Figure 4. The left images in each pair originate from the fluorescence technique, while the images on the right were captured using the trypan blue-based approach. After thresholding, the binary representation of the biofilm regions was replaced with their original grayscale values, leaving the background with a single intensity value. This adjustment was based on the intensity of the pixels to allow for a more nuanced understanding of the biofilm distribution. More details on this method will be provided in the discussion section. These results underscore the capability of the Otsu algorithm to effectively differentiate biofilms, even on very diverse substrates.

### 3.2. Image Processing—Bacterial Biofilms on Chicken

**Figure 6:**
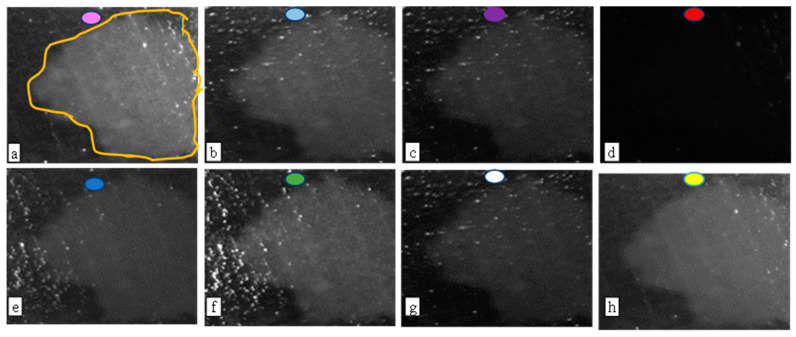
Images of a chicken-biofilm sample taken under different lights after trypan blue staining. The image containing the orange trace is the fluorescent image (standard) clearly revealing the bright biofilm regions. The colored lights that produce each image are shown at the top middle of the image. The colors are: (**a**) 405 nm UV (**b**) Cyan (**c**) Purple (**d**) Red (**e**) Blue (**f**) Green (**g**) White (**h**) Yellow.

**Figure 7:**
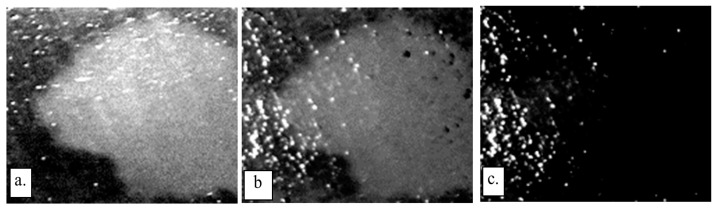
Images produced by subtracting various combinations of TB images captured under different lighting conditions. (**a**) Cyan-Purple subtraction (**b**) Green-Red subtraction (**c**) Green-Purple subtraction. The individual images are shown in Figure 6.

Chicken biofilms were stained with trypan blue to obtain TB-based biofilm regions comparable to corresponding fluorescent biofilm regions, Figure 6 shows the sample regions imaged under different colored lights. The region in the handmade contour (relatively brighter part of the image) indicates biofilms present in the fluorescent image. These images are contrast-stretched to reveal the difference between biofilm regions and non-biofilm regions. While green, cyan, and white light images appear to favor the biofilm regions, image combinations through subtraction may do better in the Otsu segmentation of biofilm regions as seen in the samples shown in Figure 7.

**Figure 8:**
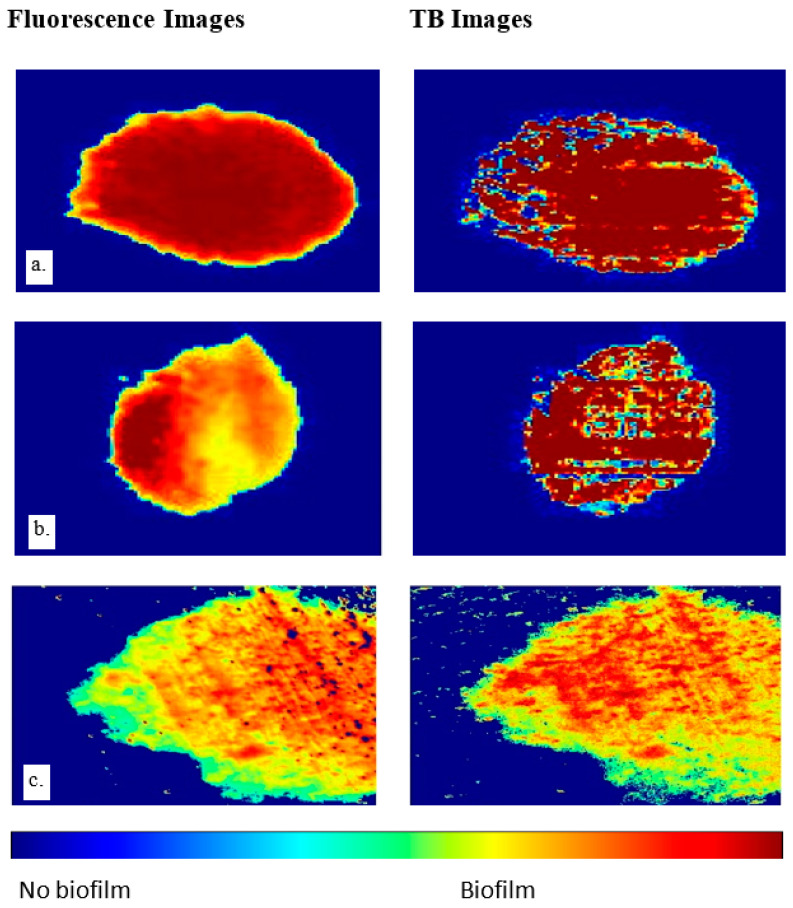
Segmented biofilm regions of 3 chicken biofilm samples (**a**–**c**), fluorescent biofilm regions (**left**) and TB biofilm regions (**right**). Quantitative analysis is in Table 1.

Figure 8 presents three distinct biofilm regions (a–c), as captured in both the fluorescent and trypan blue images. After thresholding is applied to single out pixel intensities indicative of biofilm presence, the post-processing technique used in Figure 5 was replicated. Specifically, within the biofilm regions, the binary depiction is replaced with the original grayscale values, while the background remains binary for clear differentiation. The subtler contrast between the biofilm and background pixels in these images calls for the introduction of pseudo-coloring, which enhances visibility and facilitates the distinguishing of the biofilm regions.

It’s important to note that a reliable correlation with biofilm thickness cannot be established based purely on visual inspection, necessitating a 3D biofilm analysis for accurate determination. Despite this limitation, these images provide valuable insights into the presence and location of the biofilms. Lastly, it is worth noting that Figure 8c represents the final processed version of the sample shown in Figure 6 and Figure 7, thereby illustrating the utility and effectiveness of the proposed image acquisition and processing techniques.

### 3.3. Validation

Table 1 provides the quantitative shape similarity values between fluorescent and TB images of 6 chicken biofilm calculated with Equation (1), which is a measure of how alike the biofilm regions of the two imaging techniques are. The method used to obtain the values is described in the Validation sub-section of the Experimental Setup and Procedures. Most show a high degree of similarity.

## 4. Discussion

*Pseudomonas fluorescens* biofilms are employed in this study not just because they are typical wound biofilms [3] but also because they contain fluorophores which fluoresce green under 405 nm UV light, thereby providing a validation mechanism for the proposed imaging technique. Stained bacterial biofilms on glass as shown in Figure 4 and Figure 5 confirm that TB, a live/dead cell stain is capable of binding with biofilm constituents, to map out biofilm regions on a non-living substrate. Figure 5 also demonstrates the Otsu algorithm’s capability to mask biofilm regions on a very different surface. The less pronounced biofilm formation on the glass slides, as shown in Figure 4, complicates the comparison between TB and fluorescent images. The biofilm samples appear very thin in certain areas, posing a challenge for the trypan blue staining method. The minimal thickness restricts the diffusion of the TB molecules, limiting their staining efficacy. Furthermore, the lack of adequate TB staining makes the biofilm’s representation less pronounced, which may not be sufficiently detectable by the monochrome sensor. Despite these thin biofilm regions still displaying fluorescence, TB fails to comprehensively reveal the biofilm’s complete distribution. This showcases a potential limitation of the TB technique when dealing with less established or thinner biofilm layers. However, this issue is not as significant when examining the chicken biofilms. These biofilms, well-formed on the organic chicken substrate, are successfully depicted by both the fluorescent and TB imaging methods. This underscores the applicability and utility of the TB staining technique when dealing with robust, well-established biofilms on organic substrates. While the biofilm constituents stained were not explored, studies have shown that TB will stain biofilm constituents such as polysaccharides and genetic materials [20,22,23,28,33]. However, on tissues the staining becomes more complicated, because of the dye stains both background tissues and biofilm constituents. There was, therefore, the need to explore various TB staining conditions to detect the condition that provides optimal differentiation of biofilms and tissue. A 0.04% TB concentration at 10 min staining period was found to be sufficient for this purpose. Studies have, however shown that staining conditions can be varied to obtain different staining goals [22,26,31,33]. The study also reveals that the subtraction of images taken under cyan light and purple light provides the best condition for automatic segmentation of TB-based biofilm regions (see Figure 7). It is also worth noting that the Figure 8c TB image is obtained from providing pseudo colors to Figure 7a image. The blue background is the background tissue, while the warm spectrum (yellow–red part) are the biofilm regions.

To validate the proposed imaging technique, the results were compared with fluorescence imaging to help localize bacterial biofilms on wounds at the mesoscale level [3,4,16,34]. Table 1 indicates some samples have high shape similarity values, up to 97% whether automatic thresholding was used or not, while some samples are relatively low in shape similarity e.g., 77%. This is due to several factors, such as image noise during acquisition, specular reflection, or poor biofilm formation. The current study was conducted with a relatively small dataset comprising 18 samples, including 12 glass slides and 6 chicken tissue samples. While this served as a preliminary investigation to validate the proposed method, future work will certainly focus on expanding the dataset, thereby allowing a more comprehensive analysis and robust validation of the technique.

Some of the artifacts, such as the bright spots that are particularly noticeable in Figure 8c, especially on the trypan blue images, originate from the image acquisition process. Remedial measures could include using polarizers and increasing the exposure time during image capture or employing multi-directional ambient light. As we plan to improve the methodology, a key consideration will be the reduction of glare and mitigation of uneven light distribution on the sample, which currently pose challenges to the accurate representation of biofilm thickness distribution in 2D imaging. Future work will also involve the application of 3D biofilm imaging techniques, providing a more comprehensive understanding of biofilm structures, and enabling accurate determination of biofilm thickness variations. Although the color-intensity variation in 2D biofilm images may suggest thickness variation, reliable distribution can only be ascertained through 3D analysis. The 2D images can, nonetheless, be improved and validated with 3D imaging analysis to provide a reliable representation of biofilm thickness distribution.

One of the advantages the proposed method has over the state-of-the-art fluorescence imaging is its potential applicability to both fluorescing and non-fluorescing bacteria. Moreover, the imaging parameters have been specifically tailored for bacterial biofilms, reducing the risk of false positives from fluorescing body tissues, a challenge often associated with conventional fluorescence imaging [34]. This innovative method could, therefore, serve as a means of detecting both fluorescing and non-fluorescing biofilms on wounds.

Additionally, the proposed method has potential applications in guiding surface-based treatments. For instance, the biofilm mapping can provide essential information for precision application of treatments such as Cold Atmospheric Pressure Plasma [35]. These promising developments and potential applications underscore the potential of the approach in advancing biofilm detection and analysis methodologies.

## 5. Summary and Conclusions

This study reports a new method of detecting bacterial biofilms on wounds for diagnostic or precise surface-based treatment. A novel method has been developed to detect biofilm regions on glass slides and chicken tissue. *Pseudomonas fluorescens* biofilms grown on these substrates were stained with trypan blue which stains the biofilm region differently from the substrates. Sample images were captured using a monochrome camera and cyan and purple lights (~500 nm and ~400 nm wavelengths respectively) which act as a filtering mechanism to create maximum differentiation between the background substrate and the biofilm regions. The images were processed and compared with corresponding fluorescence samples. Results show good similarity between the proposed technique and the validation method. This would, therefore, improve wound biofilm assessment as well as surface-based treatments.

## Figures and Tables

**Table 1:** Comparison of proposed method with fluorescence imaging.

Samples	Shape Similarity (Otsu Thresholding)	Shape Similarity (Manual Thresholding)
A	0.95	0.95
B	0.97	0.97
C	0.80	0.95
D	0.84	0.89
E	0.65	0.77
F	0.75	0.96

Images for samples A, B and C are shown in Figure 8.

## Data Availability

Data is contained within the article.
